# Selections and Abstracts

**Published:** 1901-08

**Authors:** 


### SELECTIONS AND ABSTRACTS.

### Some General Observations as to the Origin of Tuberculosis. How to Assist the Natural Processes in Preventing and Curing it.

By E. H. Smith, M.D., Santa Clara, Cal.

Tuberculosis is a product of civilization; civilization ultimately begets and piles up filth; filth begets, in addition to many other diseases, *tuberculosis*. In studying the history of any disease common to the inhabitants of a country of considerable extent, we always find the statement: "It is supposed that this disease was introduced among the natives by such and such people who were the earliest settlers of that country." Among the aboriginal inhabitants of any country, living in their own way, we hear nothing of tuberculosis. In the bones of such people, wherever exhumed, we find no evidence of tuberculous disease of the bones. No history is extant giving account of cough, expectoration, hemorrhages, etc., among them. "Hump-back, hip disease, white swelling," are things unknown to the original uncivilized population. They lived in relatively the same climate, ate the same food, went to sleep about the same hour, arose at the same hour, wore about the same dress year after year, decade after decade. Their habits, environment and habitation were stable for centuries. Their occupations were such as to give them abundance of fresh air and freedom from fatigue, worry, bad air, poisonous fumes, debauchery and frequent or constant excesses. They were inured to pain by traditional education, and therefore were only slightly susceptible to shock. Being virtually born and reared in the open air, they never "took cold." Not being confined in thick walled buildings and in enormous numbers, they did not suffer from lack of sewer facilities, vitiated air, polluted water, lack of sufficient sleep, enervating wear and tear of the terrific clatter of our great cities.

Contrast this with the condition of "his more fortunate brother," the civilized human being. By some caprice he began to wear more and more clothes. He built houses. He knew nothing of hygiene, and for centuries housed himself and his descendants in ill-ventilated, unsewered, scarcely lighted buildings, well calculated in every way to propagate all manner of diseases. Into these habitations he crowded his family, servants and friends. They could not talk enough in the daytime, and so he invented lights which consumed still more of the scanty oxygen in the vitiated air. He invented drinks which would intoxicate and the whole population guzzled them. He began to lay the foundation for the modern epicure, and so invented all manner of preparations for food, some of which would destroy an alligator's skin. He learned to smoke and chew and so added to the filth and unwholesome condition of his unhealthy habitation. He deteriorated in strength and resistance and began to suffer from disease. His dwellings became saturated with disease germs. Some of his wise men began to evolve "hereditary disease theories." Every time a child died of lingering disease some ancestor had to be found who had died from the same disease. Living in the infected home of a long line of filth-producing ancestors never entered into the account at all. Civilized man's wants multiplied. He began to think; thinking begets worry. He began to manufacture. Soon he began to make machines to increase the output of his manufactured products. He invented other machines to drive the first. He dug in the earth and found abundance of coal which has enabled the building up of the great trade centers, and also aided in making of them dingy, unwholesome abiding places for mankind. Men worked under ground. in blazing hot furnace rooms, in nerve-racking machine rooms and all manner of places calculated to wear out the body and mind. They lived and still exist in hovels.

The bulk of civilized humanity in our great cities do not live in as sanitary a manner as the lowest and most despised "digger Indian." They become susceptible to disease in early live if they happen to escape "the dangers of childhood." They are irritated by noises and by anxiety for the future. They inhale dust, fumes, dried germs of many sorts; eat improper food which they bolt down half-masticated; drink intoxicating and iced beverages; turn night into day, and drink and bathe in water fit only to flush sewers with. Centuries have inured them to some of these civilized hardships, so that they now live longer than might be expected. They have evolved an existence which is rapidly fatal to the barbarian. The barbarian has clung to an existence impossible to the civilized man. The barbarian left to himself is free from most of the diseases common to civilized man. Civilized man has produced a multitude of diseases more or less fatal to himself and all mankind. Among these diseases is tuberculosis. He is now blaming his ancestors for transmitting the "tendency" to this disease to him, instead of blaming their filthy way of living. Civilized man has gone on inventing, designing, scheming, gaining, until he has found to his sorrow that he must find some means to prevent death by tuberculosis from claiming so many victims. He finds that no one is immune. The medical man takes up the problem, and after more than a century of wrestling with it, has just begun to get a practical knowledge of some of the details necessary to a partial understanding of the subject. He finds that the fathers did not sin in a manner to be "visited unto the third and fourth generations," by *having* tuberculosis, but in leaving the disease behind them. Not the father or mother transmits the disease to offspring, but their filthy habitations, together with modern ways of living.

What is the problem before us to-day? Great cities, from a few decades to many centuries old, built in a most unhygienic and positively unsanitary manner. Cities that represent much of the wealth of the civilized world. Impossible to wipe them out and equally impossible to make them sanitary to any great degree. The vast majority of the civilized population vastly ignorant of how to live in a sanitary manner and not willing to learn except in the school of bitter experience. We can gradually bring about sanitary reform and do the best we can to make our cities safe places of abode, but it is a perpetual fight to hold our own against the advance of disease, with no hope of ultimately getting rid of any of the diseases now in existence.

For years physicians, actuated in part by the fantastic hope of discovering some magic cure for tuberculosis, have been wasting valuable time in laboratory and clinic, experimenting with some paltry drug, hoping against hope that they have at last found a "specific cure" for tuberculosis, and won everlasting fame. All physicians who adhere to so chimerical a fancy as curing tuberculosis by specific medication, are in the same predicament as the small boy chasing a bird with a pinch of salt, which he hopes to be able to place on the bird's tail, in the belief of a sure capture if only he can get the salt on the tail.

Civilized man has got to take the back track and revolutionize his dwellings. The wealthy man has got to build sanitary tenement houses in order to protect himself from the diseases bred in the present dirty hovels which he rents to the unfortunates compelled to live in them. All men must unite to pass and enforce proper laws of sanitation and quarantine in order to prevent the frequent occurrence and spread of disease. Preservation of health has got to take precedence over the cure of disease. The public must be taught to shun the medical braggart who promises or threatens to cure any and every disease known to medicine. More harm is done to-day by this sort of quackery than can be estimated. People must learn to keep clear of such conscienceless charlatans. Another thing which the people must learn is to preserve their religious beliefs from contamination with business or medicine. They must not let their religion become confounded with any such grotesque parody as so-called "Christian science." That the mind is a strong factor in the control of an individual in health or disease psychology amply proves. However, it is belittling and absurd for the whole human race ever to allow any such frothy vagary to gain a foothold. It is unworthy of argument, and only needs to be ridiculed to speedily die a death which shall eventually become as obscure as its origin. The present promulgators of this vagary add another pervert to the list of perverts to be considered by the student of perversity, viz: the *religious pervert.*

To prevent tuberculosis we have got to revolutionize our dwellings. Laws to compel architects to have a practical working knowledge of hygiene and sanitation before being permitted to practice their profession should be enacted. No dwelling should be permitted to be erected, in any town at least, without first having the plumbing approved by the board of health.

In curing tuberculosis, where it is possible for a cure to take place, the sick have to be prevented from commonly mixing with the well, or each other, under ordinary conditions. Certain restrictions must be placed upon them in regard to spitting, bathing, eating and drinking utensils, and use of toilet. If the digestive functions can be preserved while forcing the quantity of food, we have the greatest key to the situation. A patient with involvement of the lung only, with good appetite, perfect digestion and good elimination, has a good chance to recover. A patient with apparently small lung involvement, poor appetite, bad digestion and faulty elimination, is doomed and will invariably die, without much stay in the progress of the disease. Drugs are useful only as an adjunct to fresh air, sunshine, abundant nourishment and moderate exercise, and all backed by hygienic quarters.—*Pacific Med. Journal.*

---

### APROPOS OF BUTTONS.

There is, perhaps, nothing connected with modern civilization which shows up the hollowness, emptiness and specious pretense of some of our social customs, so much as buttons. Everybody who has any sort of a just claim to be considered anybody has heard of Sloppy "Full Private Number One in the awkward squad of the rank and file of life," who, as Dickens says, was "born to be indiscreetly candid in the revelation of buttons; and whose every button glared at the public to a quite preternatural extent." Every observing man, who has gone through the world with his eyes open, must have been struck at the appalling array of buttons which every civilized man and woman presents in their wearing apparel. Buttons that stare you out of countenance, buttons that are neither ornamental nor useful; buttons that button nothing. As mankind sinks in the scale of civilization, buttons gradually disappear, first the superfluous ones, and later the useful. The full-blown savage is untrammeled by clothes and he consequently goes about rejoicing in a single button—his umbilicus. The barbarous races make use of a few necessary buttons, and the civilized modern is distinguished, like Mr. Sloppy, by revealing in various parts of his form, "many surprising, confounding, and incomprehensible buttons."

When a man starts upon the downward path socially, the status at which he has arrived and his retrogression can be definitely gauged by the number of buttons which he can muster. The dirty, insolent, lazy, peripatetic hobo—the terror and disgust of the rural community—is generally pictured with his trousers hung by one suspender and that suspender having as a fulcrum a rusty nail. This representative of the lowest social order is as buttonless as a new-born babe and consequently is graceless and reputationless—a social pariah.

Through the wisdom and experience of ages we have come to realize that few things in this world are wholly good, and few things wholly bad; but that most things are good or bad according to the use they are put, according to the motives which underlie their use, and according to whether they are used to excess or in moderation. This is as much as to say that man himself is always capable of influencing his environment, and therefore, no one can doubt but that man can rise to a development in which he would be superior to the influence of useless buttons.

The late President Garfield once said that he never saw a red-cheeked, bright-eyed boy going to school, but what he felt like doffing his hat to him, on account of the possibilities which might be buttoned up under that homely jacket. Here seems to be a case of buttons buttoning up possibilities, and possibilities are always to be respected.

Everybody has heard of the bashful boy who while reciting his lessons got his inspiration from a certain button on his coat which he kept twirling about with his fingers. We know also that when his mischievous companions had shorn him of this helpmate, the brilliant scholar was no more able to recite than if he had been a hopeless idiot. This seems to be a case in which mental capacity depended entirely upon a single button.

All have heard of the shiftless man, or possibly we ought to say the man with a shiftless wife, who depended on a single suspender button for the covering of his nakedness. This seems to be a case in which a button occupied a very responsible position.

A few years ago a man was at work on the inside of the Washington monument near the top, when he lost his footing and down he went. In falling his clothes caught on a projecting spike and he hung suspended almost five hundred feet from the ground, his life depending upon whether his clothes could stand the enormous strain to which they were subjected. His buttons held fast and his life was saved. God bless the woman who sewed them on.

Some time ago a man at work in one of the quarries of Maine, was caught by the hook of a derrick, raised aloft, and swung out over the pit. There he hung for many long minutes, until rescued, and all that time his life depended upon the integrity of one single button. If that button had failed in its mission, this poor workman would have been hurled to certain death upon the rocks a hundred feet below.

In the light of these facts one would seem warranted in declaring that buttons have saved many lives.

Clara Barkis Peggotty, when her bosom swelled with love, and joy, and pride, was wont to bust her buttons with a crash and they went rolling about the room. Here seems to be a case in which virtues rose superior even to the constricting power of buttons.

During the French Revolution, one of the burning questions of the day was that which related to the most economical method of supplying the French armies with food. A committee, said to have been made up of the most distinguished physiologists of France, was appointed by the republic, to inquire into the exact value of gelatin as a food product. This committee formulated its conclusions in the statement that a dozen bone buttons represented a certain amount of soup stolen from the poor. Verily, great is science! This seems to be a case in which buttons by forcing themselves into public notice gave rise to a declaration that they were robbing the poor of their sustenance, and to rob the starving poor is certainly a grievous offense.

We meet people every day who are hollow, false and selfish, but who are yet maintaining an influential position in the community by going about buttoned up to the chin. Shore them of their buttons and they could not maintain their false position an instant. Such men are typified in the Tite Barnacle stamp of man, of whom Dickens said: "Mr. Tite Barnacle was a buttoned-up man, and consequently a weighty one. All buttoned-up men are weighty. All buttoned-up men are believed in. Whether or no the reserved and never exercised power of unbuttoning fascinates mankind, whether or no wisdom is supposed to condense and augment when buttoned up, and to evaporate when unbuttoned, it is certain that the man to whom importance is accorded is the buttoned-up man. Mr. Tite Barnacle never would have passed for half his current value, unless his coat had been always buttoned up to his white cravat."

Buttons have always been considered an important adjunct in maintaining discipline and to the end of upholding the majesty of the law. The officer of our home militia in giving his orders is always backed by a gorgeous array of buttons. He thunders his commands from a chest enclosed in a frock coat buttoned to the chin. Who would care a rap for his authority anyway, if it was not for his aggressive array of buttons? Could the city marshal and his efficient corps of officers uphold the majesty of the law for a moment, if they did not first stun the public by a bold, buttoned-up front? Who would have any respect for the law if it were shorn of its esoteric ceremonies, its millinery, and its buttons?

It is related of General Graut that nothing was so distasteful to him as the military frock coat. He had a great abhorrence of it. Now General Grant was a keen man and an able general; and it has been suggested that Grant may have considéred that a general could possibly rise to a position in which he did not need to depend upon a much ornamented uniform to enforce his authority or preserve his dignity.

Who ever heard of an abdominal statesman rising in his seat in the common council, for the purpose of making a speech, without first buttoning his coat to the chin? Who would care a snap for his bombast and buncombe if he did not assert himself as a buttoned-up man?

Shore all public functionaries, military officers, and civic dignitaries of their frock coats and their omnipotent buttons and what could possibly prevent this country from going to the "demnition bow-wows"? Nothing could prevent this catastrophe—nothing, but an improvement in the material which such buttons button up.

Mighty as is the power and influence of buttons in the cases already cited, the list is not yet complete, for, undoubtedly, one of the greatest triumphs of the present age is the "Murphy button." By means of this ingenious little contrivance it is possible to afford a quick and ready outlet for the bile stored up in an over-distended gall-bladder so that the dammed up gall can quickly pass out of the body *per vias naturales.*

In fact, this is about the only button that is not more numerous than it ought to be, for since we see every day how egotism, self-conceit and assurance are able to quickly force their possessor to the front, while merit, thoroughness and ability lag behind in the rear; we realize that any device which affords a ready exit for what is vulgarly called "gall" ought to come into very general use.

The sum of the goodness, kindness and unselfishness going about unbound and untrammeled, only awaiting opportunity to pour itself out for others, is more than enough to make one proud that he belongs to the human family. The amount of self-sufficiency, egotism, specious pretense and false dignity going about masquerading behind a shut-in exterior is sufficient to make one wish that some men had been born peacocks, and then they would have been expected to strut around and display their plumage. Many men are trying to pass for what they are not, and to perform duties for which they have no fitness and such hypocrites hide their defects under a fair outside. Therefore, as a rule, beware of that man, in whatever calling he may be found, who maintains his reputation by urging his button at you at every available opportunity.

The moral of this tale is obvious—don't button yourself up too tight, and if needs you must, try and button up something worth the buttoning.—*Journal of Med. and Science.*

---

### GROWING PAINS; WHAT ARE THEY? THEIR TREATMENT.

BY J. A. HALE, M.D., Alto Pass, Ill.

Owing to the frequency with which the general practitioner meets what are termed "growing pains" by the laity, and attributed by physicians to neuralgic phenomena of growth in the young, the writer desires to enter a protest against lax methods in the diagnosis.

A series of cases of "growing pains" carefully observed will exhibit besides the initial pain distinct clinical phenomena, merging one into another, and appearing at irregular intervals, and invariably diagnosed without any thought of connection between their manifestations and the prodromal symptoms of muscular spasm and pain. The fact that children suffering from "growing pains" are usually ruddy of cheek, with red lips and full pulse, bright eye, intelligent, and well nourished, and that the tendency of the pains is apparently toward spontaneous recovery, has misled many diagnosticians; and hence their oversight of certain sequelæ, which are often late in appearing.

Looking over the field of experience in a large practice among children, in both city and country, and under entirely different hygienic surroundings, the inference was drawn by the writer that "growing pains" were rheumatoid in character, were, in fact, rheumatism under different conditions from those existing in adult life, and attended with complications secondary to this disease. The clinical evidence collected by careful observations corroborates this inference, and every practitioner may be his own authority on this subject and arrive at the same conclusions.

In the presence of such a series of symptoms comprising muscular pain and spasm with a perceptible though slight rise in temperature, followed in a short time by rheumatoid complications, one naturally turns to those remedies which have proven almost specifics in their antirheumatic effect,and it is customary to administer salicylic acid or some of its derivatives. Under their use there is complete subsidence of pain and relaxation of muscular spasm; another positive proof that we are upon the right track for the eradication of the morbific factor from the system. But while we camplacently watch these good effects, does the average busy medical man consider the injurious action of such medication upon the circulatory and nervous system, which are already overtaxed in the process of growth, and by the detrimental influence of the disease. No matter how carefully we prescribe salicylic acid or sodium salicylate, the full benefit upon the disease is often not manifested unless they be administered in such doses as will exhibit the toxic effects of headache, tinnitus aurium, and depression of cardiac action. For this reason salicylic acid cannot be unreservedly employed in children. In view of the fact that this remedy has undoubtedly contributed to the fatal termination of cases under certain conditions, would it be advisable to still follow the advice of the Germans and administer large doses, especially to children?

In salicylic acid we have a drug of value; we might say a specific, but along with its merits we find well-marked disadvantages; and the problem heretofore presenting itself for solution has been, how best to rid the remedy of its defect without impairing its efficacy. Owing to the irritating action of salicylic acid upon the mucous membrane of the stomach salicylate of sodium was proposed, but it was found to exhibit the same unpleasant after-effects in lesser degree.

The demand for a non-irritating and non-toxic derivative of salicylic acid brought out a number of preparations which pass unchanged through the stomach, and are disintegrated in the intestinal canal. In some of these, however, the salicylic acid is not present in sufficient proportion to exert its specific antirheumatic action, while in others it is combined with other substances which may have a deleterious effect. A remedy devoid of these defects has finally been found, which results from the action of acetic acid anhydrid upon salicylic acid, which has been given the name of aspirin. Aspirin undergoes little or no change in the stomach, but is decomposed in the small intestines, liberating the salicylic acid in a nascent state, leaving the acetic acid to combine with alkalies present to form the beneficial compounds of sodium and potassium acetate.

Repeated trials of sodium salicylate in the rheumatism of children, or "growing pains," have convinced the writer of its efficacy to abort an attack, but have also emphasized some of its unpleasant after-effects. Further, it was noticed that it did not prevent cardiac complications or recurrences of the malady. After much search for something better, and after reading the observations of others with aspirin in the rheumatism of adult life, I was induced to try it in cases of "growing pains," with the results herewith appended, the cases being taken at random:

C. P., boy six years old, of healthy parentage and fully developed for his age. He had suffered from pains in the legs and arms with occasional muscular spasm every night for more than a week. The hygienic surroundings were good. He had been doctored with home remedies under the instructions of a "granny," but the pains became more excruciating each succeeding night. When first seen the temperature was slightly elevated, the skin dry, the pulse 120; the heart sounds muffled and faint. The child had no appetite and was peevish and cross from loss of sleep. The bowels were constipated. There was no indication of redness or swelling at any point along the extremities or around the joints. Sodium salicylate was prescribed in ten-grain doses, thrice daily. After two days there was no pain at night, but headache, tinnitus aurium and appreciable deafness, and irritable stomach. The salicylate was discontinued in the morning with instructions that the patient take nothing but nourishment. Recurrence of the pains took place that night, but the deafness, tinnitus, and headache were better the next morning. I left five-grain powders of aspirin to be given every three hours, and two cathartic granules to be taken at night. The father reported the next morning that the patient had slept well, and was free from pains; he had sweated freely, the bowels had moved, and he was playful and cheerful. Since then, three months ago, the child has enjoyed good health.

I. G., girl, 15 years old, stout, well developed, with every indication of complete puberty, except that she never menstruated. Her previous health had always been good. She was first attacked with pain, heat, and swelling in the right ankle, which disappeared the next morning to reappear in the left wrist, where it remained for two days, but left that location and affected the left ankle. The same "granny" diagnosed the case as "growing pains," the difference in character from the boy's case being due, in her opinion, to the girl's age. A brother physician was called and pronounced the affection angioneurotic edema, depending upon the approaching menstrual period. Eight days after the onset I was called to see the girl during the absence of the other physician.

My diagnosis was acute rheumatoid arthritis. (The family lived in rooms over a cellar half full of foul water.) The temperature was 101½; hot dry skin, headache, backache, constipation, no appetite, and general lassitude were present. I placed her on 15 grains of sodium salicylate immediately before each meal and at bedtime, with constitutional treatment of hematic hypophosphites, and a compound cathartic pill (improved U. S. P.) every night as necessary for the constipation. Forty-eight hours later much amelioration of the symptoms had occurred, but the patient complained of headache, tinnitus, and difficult hearing. I ordered the hyphosphites to be continued, but substituted aspirin for sodium salicylate. In forty-eight hours more the young lady remarked that she was well again. The hypophosphites were administered for some time, and she menstruated about one month after first consultation. There has been no recurrence of the pain and swelling. I am thoroughly convinced that from the onset the arthritis was of such severity that it would have required more of the salicylate to effect a cure than the patent could have tolerated.—*Pediatrics.*

---

### An Important Change in the Constitution and By-Laws of the American Medical Association.

At the annual meeting of the American Medical Association held during 1900, a committee was appointed, consisting of Drs. J. N. McCormack, Bowling Green, Ky.; P. Maxwell Foshay, Cleveland, Ohio; George H. Simmons, Chicago, Ill., to submit recommendations for incorporation in the constitution and by-laws of this association at the St. Paul meeting.

This committee made the following report, which was adopted:

"1. The delegate body shall hereafter be known as the 'House of Delegates of the American Medical Association.'

"2. The House of Delegates shall consist of not more than one hundred and fifty members, and shall be created as follows: (*a*) one delegate for every five hundred members or fraction thereof of the State and Territorial societies recognized by the American Medical Association; (*b*) one delegate from each of the Sections of the

American Medical Association, to be elected as are other officers of the Section; (c) one representative each from the U. S. Army, the U. S. Navy, and the U. S. Marine Hospital Service.

"3. Delegates representing the State societies shall serve for two years; one-half, or as near as may be, of such delegates to be elected the first year for one year only.

"4. Whenever the number of delegates exceeds one hundred and fifty there shall be such a reapportionment among the affiliated State societies as will bring the total membership of the House of Delegates below that number.

"The House of Delegates—as the Sections—shall hold its sessions daily, from 9 a. m. to 12 m. and from 2 p. m. to 5 p. m., or so much of such time as may be necessary, provided that it shall hold no session on the morning of the first day of the annual meeting, nor during the time of the General Sessions.

"6. The General Sessions of the American Medical Association shall be composed of members and delegates who may be in attendance at the annual meeting, and the time of meeting shall be 11 a. m. on the first day of the annual meeting, 7:30 p. m. on the first three days of the annual meeting, and 12 noon (or such other hour as may be agreed upon) on the last day of the meeting, which session shall be for the installation of the officers for the ensuing year and other concluding exercises.

"7. All the officers of the Association shall be elected by the House of Delegates, but no member of the House of Delegates shall be eligible to any office whose incumbent is elected by that body.

"8. No one shall be elected a member of the House of Delegates who has not been a permanent member of the American Medical Association for at least two years.

"9. The election shall take place on the morning of the fourth day of each annual meeting.

"10. No one shall be elected to any office who is not present at the annual meeting at which the election occurs.

"11. The officers elected shall be installed at 12 o'clock on the last day of the annual meeting.

"12. The membership of the Association, in addition to the delegates, shall be composed of permanent members, honorary members and associate members.

"While the committee fully appreciate the fact that its duties do not extend below the American Medical Association, nevertheless it has, in the interest of a complete organization, considered the State and local societies, and to complete this urgently required organization of the regular medical profession, offers the following recommendations to the various State and Territorial medical societies:

"(*a*) That each State society shall at the earliest possible moment appoint a Committee on Organization, to which shall be referred, with the Association's indorsement, the report of your committee, and especially that part which refers to State and county societies.

"(*b*) That each State society immediately raise funds and employ an organizer to organize the profession in its territory.

"(*c*) That the State societies unitedly agree to federate themselves in the American Medical Association, and as a preliminary to this adopt a uniform organic law in regard to certain fundamental principles, viz., to divide their annual meeting into two branches, legislative and scientific; the legislative branch to be as small as is compatible with representation from all the county societies, and to be composed of delegates elected by the county societies.

"(*d*) That membership in the county or district societies shall constitute membership in the respective State society without further dues, and that no one be admitted to membership in the State society except through county or regular district societies.

"(*e*) That funds to meet the expenses of the State society be raised by a *per capita* assessment on the county and district societies.

"(*f*) That a united effort be made to influence special societies to limit their membership to those who support the regular organization, and the semi-national and miscellaneous societies to encourage systematic organization, by covering a definite territory and also by limiting their membership to supporters of the regular organization.

"(*g*) That each State society create a permanent committee and a fund for the purpose of enforcing all medical laws in every part of its territory.

"(*h*) That each State society co-operate with the American Medical Association and with the other State societies in solving the problem now before the profession relating to medical education, medical legislation, reciprocity, licensing, etc.

"Your committee further recommends that a committee of three be appointed at the St. Paul meeting to continue, in behalf of the American Medical Association, the plans authorized in this report, and to act in conjunction with the large committee to be appointed by the various State societies. Your committee also presents herewith supplementary arguments in favor of organization. All of which is respectfully submitted."

It will be seen from the above recommendations that it will require a great deal of careful consideration on the part of the State, county or district society to formulate a plan that will harmonize and cooperate with the provisions above mentioned; however, we believe that it can be accomplished; that all societies, large and small, can be put upon a much surer and much more reliable foundation than they now occupy. While there is nothing to prevent physicians from forming lesser organizations for mutual work and benefit, yet they will have no part in the management of the organization above named. Heretofore credentials have been issued by too many societies and many disreputable men have been able to gain access to the American Medical Association, but by carrying out the above provisions carefully all of this can be avoided, and none but men of repute will hereafter be admitted to the National Association.—*Kansas City Medical Record.*

---

### QUIET EFFUSION INTO THE KNEE-JOINTS OCCURRING IN WOMEN AND YOUNG GIRLS.

Bennett (*Lancet*, February 23, 1901) in a lecture at St. George's Hospital called attention to an apparently little-noticed condition of the knee-joint not uncommon in women and young girls, which appears to be a passive effusion into the joint. It occurs but rarely in any other joint than the knee, but on one occasion Bennett saw it in the ankle.

The joints of the opposite sides are usually involved at the same time, but the effusion is generally greater on the right side. There is rarely any pain, and many of those afflicted with the effusion seem unaware of its existence. It is always, however, associated with menstrual irregularity or uterine trouble. It occurs mainly at two periods—at puberty, and when menstruation is about to cease. It may occur at any intermediate period if there is menorrhagia, excessive hemorrhage from the uterus, or great irregularity and difficulty in connection with menstrual affairs.

Although the joint contains a considerable amount of fluid, it is never tense except after superadded injury. The fluid, if the patient is standing, sinks to the lower part of the joint cavity and sometimes leads to a pouch-like overhanging of the synovial membrane at its lower anterior aspect. In the older subjects the aspects of the condition are similar, the main points being the absence of tension and heat and freedom from pain. The patients are usually anemic, but not invariably so.

After a study of several cases, Bennett advises the physician who attends a woman or girl, especially about the ages of eleven to fourteen years, to always inquire into the state of the uterine functions, and always to proceed with the ordinary surgical routine of examining both knees, although the patient may ascribe the effusion to a distinct injury of only one of them. If there be functional irregularities fluid will be found in both knees. In the absence of catamenial irregularity or uterine disorder some other cause must be sought for the condition. Any error in diagnosis can usually be avoided by noticing the character of the swelling, the existence of effusion on both sides, and the coincidence of marked menstrual or uterine trouble.

The primary treatment should be directed to the correction of the faulty functions. There should be moderate exercise and massage for the knees, combined with the healthiest of out-door lives. Splints of any kind, and an invalid's life, should be rigorously avoided. In the absence of acute symptoms from injury the condition of the knees need lead to no restriction in the exercise.

Recovery, provided the primary cause of the effusion can be cured, invariably follows unless the condition has persisted so long as to produce permanent changes in the joint. If the primary condition proves intractable the effusion will occur continuously or recur at intervals. In cases where the effusion is continuous or is constantly recurring, an increasing weakness of the knees occurs, and in the later stages, when the health becomes broken down by frequent loss of blood or great pain, edema of the legs sooner or later follows, but this has no specific meaning since it is merely the result of continuous exhausting illness.—*Therapeutic Gazette.*

---

### SOME QUESTIONS REGARDING THE DANGER OF SEPTIC INFECTION FROM CERTAIN REMEDIES AND METHODS IN COMMON USE.*

My object in presenting this paper is simply to put some questions to the Society and get the views of the members thereon.

1. In the light of modern pathology, aided by the bacteriologist, is not the promiscuous use of blisters, as ordinarily employed, a more or less dangerous practice? When the cuticle is raised and removed there is left a raw surface through which disease germs may undoubtedly gain access to the body, unless all the usual antiseptic precautions are fully observed.

I do not propose to enter into the therapeutics of blisters, but will simply ask the Society whether the advantages which they possess, if any, over the less dangerous rubefacients, in many cases at least, are sufficient to counterbalance these dangers and the suffering from pain and soreness which they generally cause? The same question may be asked in regard to wet cups. Are not dry cups less dangerous, if not oftentimes equally useful?

2. Are we not too careless in our methods of extracting teeth? How often, as a rule, do we sterilize our tooth forceps? Some twenty-odd years ago I went to a neighboring medical friend to

*Read by A. G. Blincoe, A.M., M.D., before the Brashear Medical Society at Springfield, Ky., April 16, 1901.

get an upper bicuspid tooth extracted. He lanced my gums with his pocketknife and used a pair of forceps without any cleaning at the time, as was the custom in those days, and to which I then found no fault, and after the tooth was extracted my face swelled and pained me considerably for some days. Was this pain and swelling caused by septic infection or by taking cold from bathing my feet soon afterward, as suggested by one of my friends? I can, however, recall several such cases occurring in my own practice under similar circumstances, which, so far as I know, were not followed by any bathing soon afterward at all.

3. Another practice followed by some, to which I have serious doubts as to the propriety of, is the use of the Kelly pad as commonly employed in ordinary obstetric practice. Usually, after the labor is completed, a little cold water from the rainbarrel is thrown over the rubber bag, and it is rolled up and put in a gripsack, where it remains until the doctor using it is called to the next labor case. Many of these bags have perhaps been for years in use without ever even once having been sterilized.

Gentlemen, so far as I am concerned, I would much prefer putting next to my parturient patients the clean sheets usually found in any well-regulated household.

I should like for every member and visiting physician present to give his unbiased and candid views on these questions. It may be that I am unduly apprehensive in regard to them, but I shall not fall out with any of you if you differ with me, as I recognize the fact that the prime object of our meetings is the full and free discussion of medical subjects, and, as already stated, I simply put these questions to you for the purpose of getting your opinions on them.

Another common and dangerous practice is the careless use of the uterine sound. This matter did not occur to me as being appropriate for my paper, from the fact that attention has already been called to it, but was recently suggested by a prominent surgeon with whom I was talking on the subject since the title of my paper appeared on our program.

A medical friend of mine told me several years ago of a case which resulted fatally, in which he believed death was caused by carrying matter from the vagina to the interior of the womb by means of a uterine probe or applicator wrapped with absorbent cotton.—*Amer. Pract. and News.*

---

### MUCOUS DISEASE.

Mucous disease as characterized by an irregularly occurring profuse discharge of mucus from the intestinal tract, accompanied by more or less marked nutritional disorder. The etiology is referred to an inherited tendency, either the lithemic or neurotic diathesis. It sometimes follows pertussis, but more commonly severe diarrheal diseases. There is an increased tendency to the formation of mucus in this disease, and at intervals a discharge of large masses of this substance. The child will present symptoms of malnutrition. The skin is dry and scurfy. There is frequently nausea and vomiting, the appetite is irregular. Tympanitis is present. The sleep is restless and disturbed, there is a change in the disposition of the child, loss of memory, irritability, etc. The urine is scanty and high colored, containing an excess of urates. A few days before the attack the bowels are constipated, the breath is offensive and nervous symptoms are marked. There may be dull pain in the region of the umbilicus and evidences of prostration, but there is no fever. Tuberculosis is the only condition with which mucous disease is likely to be confused, and this can be excluded by the absence of fever. Treatment should be directed toward the lithemic condition and lessening the excessive mucous secretion. Food and hygiene should receive attention. For the relief of the mucous accumulations the bitter tonics are indicated. Nux vomica is the best of these. A combination of pepsin, bismuth and strychnia is useful, as the stomach shares in the run-down condition of the rest of the body. During the period of constipation, sodium phosphate combined with sodium sulphate acts not only as a cholagogue, but tends to liquefy the mucus. A combination of hydrastis and sanguinaria acts not only as a bitter tonic, but has a direct stimulating effect on the intestinal glands. Arsenite of copper has an antiseptic action, and is sometimes used with advantage.—*Barbour, Amer. Pract. and News, July 1, 1901.*

### EPISTAXIS.

One is occasionally called to stop an alarming epistaxis, and finds himself without any instruments or drugs with which he may go to work. Some of the following expedients will stop any ordinary nose-bleed :

1. Have the patient to chew a large wad of paper or rag vigorously.
2. Cut a cylinder from a sponge; moisten well, press out all the water, tie a string to the end, and pack firmly as far back in the nostril as possible.
3. If tannic acid is at hand, it may be blown into the nostril from any tube: a pipe stem, a quill, a catheter, or even a roll of paper.
4. Ice to the back of the neck, and to the forehead.
5. Inject very warm water, if syringe be available.

For internal use, the best drugs are gallic acid, iron, hydrastis, ergot and calcium chlorid.—*Ex.*

---

### TREATMENT OF HEMORRHOIDS.

Dr. J. P. Tuttle, as noted in *Amer. Med.*, believes that in many acute cases of internal hemorrhoids, local and general measures should be resorted to rather than operative procedures. Cold water enemas once or twice a day are of great benefit in order to produce an easy movement of the bowels and to contract to some extent the blood-vessels. Injections of mild non-irritating astringents, such as the fluid extract of krameria, fluid extract of hamamelis, or Pinus Canadensis, will have a very soothing and curative influence. Suppositories of ichthyol, tannic acid and belladonna are of great benefit, especially if there is an eroded condition of the parts. Resinous cathartics, such as podophyllin, aloin, gamboge, etc., irritate the parts and should not be used, Small doses of saline laxatives, especially sodium phosphate, before breakfast, followed after breakfast by a cold enema, will have splendid effect upon the liver, intestine, and hemorrhoids.

